# ESDM intervention in severe preschool autism: an Italian Case report, psychological and social medicine reflections

**DOI:** 10.1186/s13052-024-01626-9

**Published:** 2024-04-04

**Authors:** Rosaria Ferrara, Felice Damato, Leonardo Iovino, Flavio Marti, Roberto Latina, Costanza Colombi, Pasquale Ricci

**Affiliations:** 1 Department of Anatomy Histology, Legal Medicine and Orthopaedics, Sapienza University of Rome, Rome, Italy; 2 “Parthenope” University of Naples, Naples, Italy; 3 Department of Health Professions, A.O. San Camillo Forlanini, Rome, Italy; 4 Nursing Science, Department of Health Promotion Science, Maternal and Infant Care, Internal Medicine, and Medical Specialities (PROMISE), University of Palermo, Palermo, Italy; 5 IRCCS Stella Maris Foundation, Pisa, Italy

**Keywords:** Autism, ESDM case report, Public health, Developmental psychology, Psychological assessment, Social medicine

## Abstract

The goal of our contribution is to discuss a preschool intervention based on the Early Start Denver Model and the use of the main tools for the detection of adaptive behaviour in cases of autism: Vineland, ABAS. Case presentation: the work is the presentation of a clinical case that has benefited from an intervention with the Early Start Denver Model methodology for the benefit of a child with socio-cultural and economic disadvantages. This early intervention, in a child of 36 months, which followed the diagnosis, was possible thanks to the intervention of many third-sector organizations which allowed this child, with a serious autism profile, to receive an evidence-based intervention for free. At the beginning of the intervention, the child presented a diagnosis of severe autism with absence of gaze, vocalizations and other communicative impairments. The level of motor clumsiness was also quite high, as were stereotypies. Conclusions: Research has shown the usefulness of intervening in this area with an early assessment and/or diagnosis and immediate intervention; however, public health services are not always able to maintain this pace. Our contribution therefore shows on the one hand the evidence of the improvements achieved by the child despite the low intensity of the treatment, and on the other hand, demonstrates the total versatility and adaptability of the Denver Model to the Italian context. In our conclusions, there are also some reflections on the tools used to measure adaptive behavior which seem to have a number of limitations and criticalities.

## Background

 Autism spectrum disorder (ASD) is a neurodevelopmental disorder that involves deficits in social skills and verbal and nonverbal communication, as well as the presence of stereotyped behaviours and narrow interests [1] with difficulties in executive functions as well [2]. Autism has an incidence of about 1:77 in Italy [3] and results in high costs for the National Health System and families. Autism is a condition, not a disease therefore there are no cures but treatments, and it has reconfigured itself over the years, making its diagnosis more and more specific and far removed from the realm of psychosis [4]. The onset of autism is in early childhood [5], and involves the subject in its entirety, with repercussions also in the subject’s inflammatory system [6].

There is updated evidence which supports how the earliest intervention, after the arisen of behavioural untypicalities, allows the child an immediate development of those areas that would otherwise be increasingly affected by autism [7]. The presence of an autistic child within the family results in a significant reduction in quality of life and an increased risk related to the development of mental disorders among parents or caregivers. Therefore, the early invention not only improves the living conditions of the child but also helps the caregiver’s well-being. Studies show that intervention initiated immediately after diagnosis in the first three years of life leads to better outcomes than interventions delivered later [8]. However, in the Italian context, it is not so easy to access therapies just when this window of neuronal plasticity requires us to do so. There are long waiting lists to enter public service, and parents cannot always afford private intervention. Therefore, it’s useful to consider that lower economic affordability corresponds to a greater need to enter the public health service.

So, it is necessary to think about an early intervention that also involves caregivers equipping them with techniques and strategies that they can use at home, as well as at the school where the child attends. In this way, a series of figures, in addition to the therapist, are to be formed who will begin to interact with the child following the principles dictated by specific training. It is precisely for this reason that in this article we recount our intervention based on the ESDM (Early Start Denver Model) in different pragmatic contexts that allowed us to train parents and school workers as well as to carry out an intervention on the child himself. ESDM is an intervention of supported effectiveness [9] developed for children diagnosed with ASD or at risk in preschool. In addition to being evaluated in the research context (experimental efficacy), several studies in the Italian area context have shown its clinical efficacy [10, 11]. ESDM is presented as a context-adaptable intervention in that it can be delivered with the child, with the child and a parent (Parent-ESDM), in the school setting, and via computer. In research by Dawson et al. [9] it was found that a group of 24 children who had received ESDM therapy for 20 h per week with a 1:1 modality combined with parent-ESDM had greater improvements in developmental domains after two years of intervention than children who had received other type of therapy with similar intensity. It is possible to engage parents quite effectively with ESDM therapy as well as apply these strategies to the school through small group interventions [12].

### Characteristics of the ESDM and the Italian context

The ESDM [13] Is a comprehensive, manualized intervention program for children with ASD between the ages of 12 and 60 months [9] and is an approach based on strategies of Naturalistic Developmental Behavioral Intervention (NDBI) [14]. This method envisions learning taking place within naturalistic therapy settings that leverage the child’s preferences and play activities [15]. In the ESDM intervention, there is no external reinforcement, but the joint and naturalistic activities are inherently rewarding for the child. This implies that an earlier application of this method results in a wider margin of improvement in the child’s functioning. These margins of improvement were found to be even wider when associated with good imitative skills, in playing with objects and joint attention observed at the beginning of therapy [16]. Increased intervention intensity has been cited as another influencing factor by several researchers [17]. Another predictor of a good outcome of ESDM therapy is the development of social-communicative gestures, which are now associated with language development such that limited social use of gestures is a symptom of an underlying early-onset social attention deficit, leading to a deprivation of the social learning experiences necessary for the acquisition of verbal and nonverbal skills [16].

It is interesting to think about the application of ESDM to the Italian context. The diagnosis of autism turns out, even today, to be very late, Scientific evidence has now demonstrated the need for early intervention, capable of affecting the developmental trajectory, but in Italy as well as other countries, children arriving at the services around the age of fiveand usually can’t benefit from interventions aimed at ASD [18, 19]. This lack, which may vary from region to region, is very often made up for by Associations and Third Sector organisations whose mission is bio-psycho-social wellbeing, and they work at this purpose through the employment of many professionals and volunteers. These efforts make a series of cures and/or treatments more accessible, not only in terms of time saved but also economically. At present, the Italian Health Service is still unable to cope with the complexity of autism, which ranges from very early childhood to adulthood. however, it has already deployed various forces and, in the Italian context, the important role of nurseries, kindergartens and schools of all kinds and degrees, which are always and for all subjects inclusive, should not be underestimated. The ESDM has been used and validated within various Public Health systems, demonstrating its practicability, adaptability to the Italian Health System and effectiveness. The use of ESDM in Italy is characterized by its adaptation to the highly inclusive school context, which makes this country an example for the treatment of disability. Therefore, there is scientific evidence of how the use of ESDM even at low intensity produces clear improvements in children with autism. The intervention protocol discussed here is the same one used [20] by the named regional program S.F.I.D.A. (Screening, Friuli Venezia Giulia, Intervention, Diagnosis, Autism). The project, carried out within the public health system of a region in north-eastern Italy, compared two groups of children treated with the ESDM and TAU implemented at very low intensity (2 h per week) through the first year, investigating the feasibility and effectiveness of the ESDM provided in community settings. The methodology used in this article, therefore, has already been used and published in scientific circles.

## Case presentation

The intervention analysed in this paper was conducted over nine monthsperiod on a group of preschoolers who received a free ESDM intervention; the child described in this study was included in the initiative presented above. Consent to the publication has been obtained from the parents of the child, the data reported protect and guarantee the anonymity of the subject. The intervention included one hour per week of 1:1 ESDM therapy, one hour per week of parent-ESDM therapy, the possibility of a monthly meeting with the school, and constant weekly supervision by Dr. Colombi.

This approach aimed to provide comprehensive support and ensure the child’s progress. Meetings with schools have been a means of sharing the therapy’s goals, as well as strategies and techniques for achieving them, with teachers. ESDM emphasizes involving teachers, child welfare workers, and related health professionals in a multidisciplinary team [21]. However, in the specific case we’re discussing, the child had not yet been placed in a school setting, so school meetings were not conducted. Nonetheless, it was crucial to involve the mother in ESDM therapy to share goals and strategies. An essential goal of ESDM is to empower parents to implement intervention techniques throughout the day and across various natural environments and activities, rather than limiting them to designated therapy sessions. This approach potentially increases the amount of intervention the child receives [22].

The primary goal of the proposed project is to enhance early access to intervention by tailoring the delivery to the child’s life context and optimizing the use of available resources. The case presented involves a child with severe nonverbal autism, characterized by a lack of gaze and pervasive motor stereotypies, such as tiptoeing and object shaking. The child follows a regular sleep–wake cycle but experiences difficulty with chewing due to strong food selectivity, primarily consuming milk, and soft biscuits. Currently, the child has not been enrolled in any school setting and resides with his mother, father, and grandmother. He predominantly spends his time with his non-working mother. The mother experiences profound shame concerning the child’s challenges and sees him as lacking abilities. Additionally, the child exhibits self-directed vocalizations throughout the day while engaging in self-stimulatory behaviors or observing his hands. There are no reported cases of autism or related conditions within the family.

### Measures

Diagnostic criteria for autism include a range of difficulties in social communication and reciprocity, as well as the presence of several restricted and repetitive behaviors and sensory impairments [23]. However, in addition to the difficulties just listed, other inabilities in daily life affect adaptive behavior [24]. Adaptive actions relate to the chops demanded to perform tasks of day to day living related to real life capability. dimension of adaptive functioning has taken on added significance because of its adding part in the opinion of intellectual and experimental disabilities, in determining eligibility for technical services and programs, in treatment planning and monitoring, and in the assessment of functional impairment in exploration and opinion [25]. The instruments used in this study go in the direction of measuring after an ESDM intervention the characteristics of adaptive behavior through the instruments designed to detect it. Such instruments are the Vineland [26] and the ABAS [27]. The Vineland is the most widely used tool for quantifying difficulties in adaptive behavior in the areas of socialization, communication, and daily functioning [28]. The Vaineland II (Vineland Adaptive Behavior Scales-Second Edition) can be used from birth to 99 years of age, and its administration yields a statistically “normalized” score similar to the Wechsler scales. Specifically, it aims to measure adaptive behavior in the domains of Communication, Skills of Daily Living, Socialization (in individuals from 0 to 90 years of age) and Motor Skills (in individuals from 0 to 7 years of age and from 56 to 90 years of age). The assessment of adaptive behavior is necessary for the diagnosis of intellectual disability disorder and, in accordance with the DSM-5, for the evaluation of the severity of impairments. The Vineland II has been used in clinical research settings with different populations [29] such as Fragile X, Autism and Down Syndromes [30].

ABAS The ABAS-II Adaptive Behavior Assessment System. 2nd edition is a questionnaire consisting of eleven skill areas organized into three general domains: conceptual, practical, and social [31]. The optional Work skill area available for adolescents and adults was not completed in this study. The composite and domain scores are standard scores with a norm referenced mean of 100 and a standard deviation of 15; skill area scores have a mean of 10 and a standard deviation of 3. Items are rated on a four-point scale ranging from 0 = Is not able to 3 = Always/almost always.

ADOS The ADOS-2 is a semi-structured, standardized assessment of communication, social interaction, play, and restricted and repetitive behavior. It presents various activities that elicit behavior directly related to a diagnosis of ASD. By observing and coding behavior from 0 (absence of symptoms) to 3 (presence of strong symptoms), the examiner can obtain information that informs diagnosis, treatment planning, and educational placement. The ADOS is one of the few standardized measures administered to a child by a trained professional that allows for direct observation of behaviors characteristic of ASD. Many people who utilize the ADOS as part of their assessments view the administration and the measure’s capacity to identify ASD-related behaviors as positive. The most commonly reported disadvantages of the measure were the tendency to over-classify children who have other clinical disorders, the cost of the test kit, and the time it takes to administer the measure [32].

## Results

The instruments described above were administered to the child producing the following results:

Before presenting the table of results, it is useful to point out some features of the present study. First, the ADOS tests for autism spectrum diagnosis were administered only during the first sessions. While ABAS and Vineland II, which assess adaptive behaviors, were administered during the first session and after nine months of ESDM treatment. Considering the objectives of the present case report, the authors focused the study on tests assessing adaptive behaviors.

**Table:** 

ADOS	SCORE
AS + CRR	26
PC	10

### ABAS-II

The results provided by ABAS-II consist of four scores of which one describes the general adaption (GAC), and three are related to different adaptive domains as Conceptual (DAC), Social (DAS) and Practical (DAP). For the present case report, all the raw scores were collected both in the first and after nine months of treatment using the parent/caregiver evaluation sheet. Additionally, the weighted and composite scores were computed using the Italian adaption of ADOS-II [33]. Table 1 shows all the subject’s ADOS-II scores.

**Table 1:** ABAS-II valutation

***ABAS-II***	***T0***	*T0raw*	***T1***	*T1raw*
***GAC*** *General Adaption*	**42**	*17*	**42**	*18*
***DAC*** *Conceptual Adaptive Domain*	**50**	*7*	**43**	*4*
***DAS*** *Social Adaptive Domain*	**52**	*3*	**52**	*3*
***DAP*** *Practical Adaptive Domain*	**48**	*6*	**57**	*12*

T0-T1 9 months’ time span, T0 first administration, T1 Administration after 9 months of treatment

The results collected during the first administration (T0) showed that general adaptation and the three domains assessed by the ABAS-II were extremely low compared to the mean score. This means that considering the specific sensitivity of ABAS-II, the child before the ESDM therapy had poor adaptive functioning in all assessed domains and their respective subdomains, including areas such as communication, self-control, socialization, self-care, and use of the environment, etc.

However, weighted in T1 scores showed that for the three domains only the Social domain (DAS; play/leisure and socialization) was extremely below average, while the Conceptual and Practical index showed to be closer but still below average. Indeed, the GAC score turns out to be the same as calculated during T0 but, looking at the other domains, the DAC and DAS indices are lower than the first assessment while the DAP (which assesses Self-Care, Home/School Life, Health and Safety, and Use of Environment) appears to have improved although still below average. Moreover, weighted scores DAC and DAP fall within the “borderline” and “average” ranges, respectively. Considering the starting setting shows that ESDM therapy causes measurable effects during the nine months of treatment.

### Vineland II

Table 2 shows the scores obtained from the Italian norms of the structured interview Vineland-II. This test, compared with ABAS-II, assesses the adaptive behavior considering four major domains (derived scores): Communication, Daily living skills, Socialization and Motor skills. Each main domain is respectively composed of specific sub-domains (for example the sub-domains Receptive, Expressive and Written help to the interpretation of the Communication domain) that are computed under the form of a v-scale which describes the individual relative level of functioning compared with the group of subjects of the same age.

**Table 2:** Vineland II valutation

*Vinerland II*	*T0*	*T1*
*Sum of domain standard scores*	*168*	*175*
***Full scale***	***33***	***33***
***Communication***	***26***	***28***
*Receptive*	*1*	*2*
*Expressive*	*1*	*1*
*Written*	*15*	*12*
***Daily living skills***	***45***	***39***
*Personal*	*1*	*1*
*Domestic*	*10*	*9*
*Community*	*8*	*7*
***Socialization***	***40***	***43***
*Interpersonal Relationship*	*2*	*6*
*Play and Leisure Time*	*2*	*2*
*Coping Skills*	*10*	*10*
***Motor skills***	***57***	***65***
*Gross*	*9*	*9*
*Fine*	*8*	*8*

T0-T1 9 months of time span, T0 first administration, T1 Administration after 9 months of treatment

The scores shown in Table 2, like those in ABAS-II, are labelled T0 and T1, which have the same meaning as the indexes described in the previous paragraph. Pre and post administration showed that after nine months of treatment, the domains of Communication, Socialization and Motor Skills appeared to improve. all major scales, looking for their inclusion in the adaptive level ranges, were found to be included in the low range in both T0 and T1. While daily living skills scores were lower in T1 than in T0. In terms of the sum of domain-specific standard scores, T1 (175) shows an increase of 7 points compared to T0 (168); despite these data, full-scale values, calculated by converting the sum of standard scores, were the same in both T0 and T1 (33), considering the norms, the full scale is invalid because the profile is too inhomogeneous. The comparison of v-scale values for subdomains shows a likely protective effect toward the child who is not penalized by the age-equivalent Vineland II scores that show greater statistical sensitivity for such a highly impaired subject as early as preschool age. While most of the subdomain scores (Receptive, Expressive, Domestic, Community, Interpersonal Relations, Play and Leisure, Gross, and Fine) are among the “low” values in the ranking, the remaining scores (Written, Domestic, and Coping Skills) fall in the “moderately low” score ranges. Only written subdomain resulted to be adequate in T0.

## Discussion and conclusions

ASD is a lifelong persistent disorder. Assessment of adaptive behavior is important to identify the severity of deficits in different areas and to plan goals in clinical, family, and school settings [34]. As pointed out in the literature, assessment of adaptive behavior can be particularly difficult for individuals with lower skill levels. The main problem is linked to the floor effect, which produces fat profiles. Consequently, strengths are often concealed, and weaknesses appear predominant. Then Vineland II (VABS-II) shows a greater sensibility to the critical areas as opposed to ABAS. As far as ABAS is concerned, the Major improvement we have in the Practical domain (PRAC; encompassing Self-Care, Home Living, Community Use, and Health and Safety), while in the assessment with the VABS II it is the Daily Living Skills domain that doesn’t show any improvement, differently from indexes referred to the other domains which show improvements. This effect has already been investigated in the literature, these comparisons of similar skill subdomains/areas, the VINELAND-II yielded significantly higher scores for six of the seven comparisons. The only subdomain/skill area which yielded a contradictory finding was Play and Leisure which was significantly lower for the VABS-II compared to the ABAS-II. Overall, these findings indicated that the degree of impairment in adaptive levels varied considerably across the instruments. In general, the VABS-II yielded higher scores than the ABAS-II [35]. Wanting to discuss the results from a broader perspective, we can state that the effects of ESDM are observable in the present case study. Similar effects were observed in a recent Japanese study, in which it was shown that even after a relatively short duration of ESDM intervention [36], less than one year, children show improvements in language and socialization. This study demonstrated that ESDM intervention could reduce the severity of social impairments and language delay as assessed by the VABS, and Practical domains rated by ABAS II. Deficits in social communication and social interactions are the core features of ASD. The results suggest that the ESDM intervention protocol (75 min per session and once a week) would show a reduction in the severity of some clinical symptoms of ASD. It is necessary to point out how some factors, which show improvement within the ESDM worksheets, are not detected in the ABAS and VABS assessment protocols. These factors are eye contact, increased hetero-directed vocalizations, and decreased stereotypies.

Autism is an extremely varied condition, and each individual clinically differs from the others according to symptoms, duration, and impaired areas [37]. A major watershed in autistic variability is IQ, and about 44% of autistic individuals have IQs in the normal range or above [38], in addition to cognitive aspects the phenotypic variability of autism, which is constantly reconfiguring, is also related to language abilities and adaptive behavior [39, 40]. A low cognitive profile appears to be predictive of poor adaptive behavior; however, severe difficulties may be present even in those with good IQ [41]. Thus, IQ appears to be predictive of low adaptive behavior for those with low IQ, while for those without deficits other aspects such as autism symptoms, verbal memory, and language [42]. The case report here discussed is of particular interest not only for clinical and developmental psychology but also for social medicine. Social medicine addresses the overall well-being of individuals in society, taking into account legal aspects related to disabilities and the recognition of fundamental rights [43, 44]. Therefore, for all the aspects already highlighted in the preface, including the connection between economic disadvantage and late intervention, the question appears to be of medical and social interest.

In conclusion, the clinical case presented highlights the discrepancies between the two most widely used instruments for assessing adaptive behavior; however, both instruments, with the due limitations previously argued, showed improvement in the subject 9 months after the start of treatment. Our case appears to be in line with previous studies that have demonstrated the effectiveness of ESDM in improving cognitive and language abilities, as well as social skills. To evaluate the effect of the ESDM protocol more adequately with detectable results from the ABAS and Vineland, the treatment should be extended to a significantly larger population and for a significantly longer time.

## Data Availability

All data generated or analysed during this study are included in this published article.

## Abbreviations

ASD: Autism Spectrum Disorder
ESDM: Early Start Denver Model
ADOS: Autism Diagnostic Observational Schedule
ABAS: Adaptive Behavior Assessment System
